# Writing an effective manuscript: Editor's perspective

**DOI:** 10.4103/0253-7613.43157

**Published:** 2008-08

**Authors:** Mira Desai

**Affiliations:** Assistant Editor, Indian Journal of Pharmacology, Department of Pharmacology, B. J. Medical College, Ahmedabad - 380016, Gujarat, India. E-mail: desaimirak@yahoo.co.in

Writing a research article and getting it published is an appropriate and acceptable means of reporting new information to the scientific community. It is also the stepping stone for the professional career of a research scientist. However, scientific writing is not all that easy! The journey of scientific writing starts with planning and execution of research. Often the experience is not satisfactory. An attempt to publish the research results in a reputed journal is an arduous task for postgraduates and junior faculty members. The quality of manuscripts submitted at Indian Journal of Pharmacology (IJP) and the efforts made in revising them have been a matter of frequent discussion among the editors. We would like to share some of these thoughts which may help the prospective authors. 

Rarely a manuscript submitted is ‘too good’ to be accepted immediately or ‘too bad’ to be rejected obviously. Most of the manuscripts stand in between ‘too good’ and ‘too bad’. Although peer reviewers provide the scientific opinion about the manuscript, the final decision of acceptance or rejection depends upon the editors. Besides the scientific content, the editors are also concerned about few ‘little things’ which ultimately shape a manuscript for publishing. What are these ‘little things’ that editors look for? The format, novelty of research subject, brevity, language, clarity, message and quality of presentation do matter for the editors.

At submission, the editors consider the relevance of the manuscript for the journal and send it to the appropriate reviewers. Articles not relevant to the journal's area of interest are obviously sent back without any external review. While all the original research articles, reviews etc. are always sent for external review, those like case-reports, letter to editor are often reviewed in-house. We have often observed manuscripts having either a long, overstated title along with adjectives and abbreviations or having an unclear, ambiguous meaning. The title should be simple but eye-catchy, brief but convey essence of the study along with important key phrases. The title and the structured abstract should draw the attention of the prospective reader. Avoid including points in the abstract which have not been mentioned in the text.

Most of the research articles submitted to IJP follow the IMRAD[1] format and style but the copyright and authors declaration forms are often missing. No article is rejected only because it does not follow a journals' prerequisite. However, the missing items delay the publication process. Following a recent issue of the journal for the checklist and instructions will certainly help the authors. Secondly, the amount of information included in each section of IMRAD is extensive at times which makes the job tedious for the reviewers and editors. Long and detailed introduction, too many and too old references, unusual length of the article and failure to summarize briefly suggest a poor quality of work and its presentation. The journal space is limited and this necessitates the restrictions on word length (maximum 3200), references (maximum 25) and tables/figures (5–6).

Further, the editors always look for the novelty of the work. If the research area is novel and of current interest or is involving an uncommon method, unusual study design or an uncommon topic, it certainly generates interest. Editors always look at the manuscript from the readership point of view and judge whether such an article published in the journal, will be useful and interesting to the readers or not? An interventional study or a study providing additional information on economic impact or quality of life would definitely generate interest among readers and editors as compared to the conventional reports. Due consideration is given to a manuscript even with uninteresting or negative results if the topic or methods are instructive and novel. A clear presentation helps reviewers and editors to understand what has been done and this saves their time. The editors match the objectives with concluding remarks and the discrepancies are pointed out, if any. The speculations beyond the results (findings at cellular level and use in human beings) should be avoided.

English is an important barrier in writing a manuscript especially if the formal education of the authors has been in vernacular language. However, editors always look for brief and simple words and not really the linguistic expertise. Use of long and complex sentences should be avoided. All that is required is to write simple English with an attempt to inform and educate the readers rather than to impress them. A manuscript with poor language, spelling mistakes or grammatical errors will probably never cause a rejection. But, the amount of revision, rewriting required for accepting that paper will always be considered in making the decision to accept or reject.

Typographical errors and spelling mistakes are considered unprofessional by reviewers and editors. The authors need to take extra care and be consistent with the use of capital letters, abbreviations and spellings. One should also avoid using abbreviations and adjective in the title. The word ‘significant’ should not be used loosely and if used it should be followed by *P*  value. 

A manuscript which is accurate and concise with a clear message conveyed in a crisp way certainly impresses the editors. Several other factors may be considered by editors before accepting or rejecting a manuscript which is beyond the scope of this editorial to discuss. The ‘little things’ mentioned above are equally important for a good manuscript. These skills need to be cultivated, refined and improved by writing, rewriting and carefully examining the good quality paper paying particular attention to their style rather than just reading the contents. Introduction of scientific communication skills in the post graduate curriculum may help the beginners in this regard.
